# Intrauterine synechiae after myomectomy; laparotomy versus laparoscopy: Non-randomized interventional trial

**Published:** 2015-03

**Authors:** Zahra Asgari, Leili Hafizi, Rayhaneh Hosseini, Atiyeh Javaheri, Hathis Rastad

**Affiliations:** 1 *Department of Gynecologic Laparoscopy, Arash Women’s Hospital, Faculty of Medicine, Tehran University of Medical Sciences, Tehran, Iran. *; 2 *Department of Obstetrics and Gynecology, Imam Reza Hospital, Faculty of Medicine, Mashhad University of Medical Sciences, Mashhad, Iran. *; 3 *Department of Obstetrics and Gynecology, Faculty of Medicine, Shahid Sadoughi Hospital, Shahid Sadoughi University of Medical Sciences, Yazd, Iran.*; 4 *Research Development Center, Arash Women’s Hospital, Faculty of Medicine, Tehran University of Medical Sciences, Tehran, Iran.*

**Keywords:** *Intrauterine synechiae*, *Uterine myomectomy*, *Laparotomy*, *Laparoscopy*, *Hysteroscopy*

## Abstract

**Background:**

Leiomyomata is the most frequent gynecological neoplasm. One of the major complications of myomectomy is intrauterine adhesion (synechiae).

**Objective:**

To evaluate and compare the rate and severity of synechiae formation after myomectomy by laparotomy and laparoscopy.

**Materials and Methods:**

In this non-randomized interventional trial, hysteroscopy was performed in all married fertile women who had undergone myomectomy (type 3-6 interamural and subserosal fibroids) via laparotomy and laparoscopy in Tehran’s Arash Hospital from 2010 to 2013. Three months after the operation, the occurrence rate and severity of intrauterine synechiae, and its relationship with type, number and location of myomas were investigated and compared in both groups.

**Results:**

Forty patients (19 laparoscopy and 21 laparotomy cases) were studied. Both groups were similar regarding the size, type (subserosal or intramural), number and location of myoma. The occurrence rate of synechiae in the laparoscopy and laparotomy group was 21% and 19%, respectively; showing no significant difference (p=0.99). Among all patients, no significant relationship was found between the endometrial opening (p=0.92), location (p=0.14) and type of myoma (p=0.08) with the occurrence rate of synechiae. However, a significant relationship was observed between myoma’s size (p=0.01) and the location of the largest myoma with the occurrence of synechiae (p=0.02).

**Conclusion:**

With favorable suturing methods, the outcome of intrauterine synechiae formation after myomectomy, either performed by laparotomy or laparoscopy, is similar. In all cases of myomectomy in reproductive-aged women, postoperative hysteroscopy is highly recommended to better screen intrauterine synechiae.

## Introduction

Smooth muscle cells of the uterus can be the origin of benign tumors such as uterine leiomyomata (fibroids), which is the most frequent gynecological neoplasm. It has been estimated that around 20-50% of all women experience uterine myoma during their life time. Their incidence increases as the woman’s reproductive life approaches its end (1). Approximately 25% of reproductive-aged women show such clinical signs and symptoms (2). Myomas can be categorized based on their position towards the uterine wall as subserosal, interamural and submucosal types. Most myomas are asymptomatic with no need for treatment. The clinical presention, however, may have the following forms: vaginal bleeding, abdominal or pelvic mass, urine signs and symptoms, disgestive complaints, infertility and pelvic pains. They also increase in size during pregnanacy and usually lead to pregnanacy complications such as abortion, preterm labour, and bleeding after birth (3). 

The definitive treatment for myoma in a fertile woman is surgery; whereas medical treatment is not appropriate for those who want to maintain their fertility. It is specially inappropriate in large myomas (4). Therefore, in those patients who insist on preserving their fertility, myomectomy is the gold standard surgical approach (5). Laparotomy, laparoscopy, or hysteroscopy are different techniques by which myomectomy can be performed. Hysteroscopic myomectomy is limited to the treatment of submucosal fibroids. On the other hand, laparotomy or laparoscopy is required when there are symptomatic intramural and subserosal fibroids, needed to be surgically removed. However, abdominal myomectomy (AM) can lead to significant morbidities including excessive blood loss, a high rate of infection, and postoperative adhesions (6).

Myomectomy through laparoscopy, which was first introduced in 1979 by Semm *et al* is a minimal access approach (7). It was designed to answer the needs of women who wish to maintain their uterus and fertility. Three different prospective randomized studies have compared the outcomes of abdominal and laparoscopic myomectomy (LM) (8-10). These trials indicated that LM leads to shorter hospitalization and a more rapid recovery. Additionally, very fewer incidences of postoperative pain, fever, analgesic use, and anemia has been observed in LM rather than AM cases (11). It has also been reported that the occurrence rate of adhesions is considerably less in the laparoscopic approach compared to abdominal procedures (12).

Intrauterine trauma can cause a condition called Ashermen’s syndrome or "uterine synechiae" or intrauterine adhesions which are defined as abnormal fibrous connections that join tissue surfaces in abnormal locations (13). They mostly (more than 90% of cases) result from curettage, however, manipulations of a nongravid uterus, such as dilatation and curettage, metroplasty, myomectomy, and hysteroscopic surgery can also lead to synechiae (14-16). The clinical presentation of those afflicted by intrauterine adhesions is usually one of the following: infertility, menstrual cycle disorders, repeated pregnancy loss, or abnormal adherence of the placenta (15). The gold standard for diagnosis is hysteroscopy (17). In a successful conservative treatment of fibroids, the functional preservation of the uterus must be considered as crucial as tumor removal or symptoms relief. Surgical trauma to the endometrium which is a usual byproduct of trans-mural myomectomies can cause adhesions (18, 19). According to the modified classification of the European Society of Gynecological Endoscopy (ESGE), intrauterine adhesions can be categorized into three types:

Mild: Filmy adhesions that would cause partial or complete uterine cavity occlusion.

Moderate: Fibro muscular adhesions which are covered with endometrium.

Severe: Adhesions which are merely composed of connective tissue (20).

Although the underlying factors that lead to adhesion formation are still mostly unexplained, trauma to the endometrium is commonly considered as a major factor. Trauma to the basal layer of the endometrium can trigger the development of intrauterine scars which lead to adhesions (18). One of the other risk factors causing intrauterine adhesions is myomectomy through laparotomy (21). It is speculated that infection or inadvertent closure of the uterine cavity may play a role in adhesions development (22). 

However, diagnostic pitfalls and low awareness can result in an underestimation of intrauterine adhesions following myomectomy (23). Based on our current knowledge, we would strongly recommend a strategy that would both minimize the surgical trauma and identify the high-risk cases. Moreover, early hysteroscopy diagnosis is probably the best means for secondary prevention and treatment of postoperative (after myomectomy) intrauterine synechiae (18, 24). 

The purpose of this study was to evaluate the frequency and severity of uterine synechiae following myomectomy via the laparotomy and laparoscopy approach; their comparison besides determining the relationship between intrauterine adhesions with the myoma’s size, location, and type; and also with endometrium opening during surgery.

## Materials and methods

In this non-randomized interventional trial, all of the married fertile women who had undergone myomectomy (type 3-6 intramural and subserosal fibroids) in Tehran’s Arash Hospital from 2010-2013 were enrolled. Because this study was a new research in Iran and we could not anticipate patient’s cooperation about complete follow-up, so we preferred the non-randomized trial. All myomectomy patients having the inclusion criteria and in the mentioned time period were studied; so the sample size was determined based on the number of patients visiting the clinic during this time period. A written informed consent was obtained from each participant prior to study entrance. 

All surgeries were performed by two gynecological laparoscopists with the same technique. None of the patients received analogous GnRH before the operation and all of them underwent surgery in the follicular phase of their cycle (except for those with metrorrhagia). Those patients, for whom laparoscopy was contra-indicated due to the myoma size or other parameters, were excluded from the study. The decision whether to have laparoscopy or laparotomy was made solely by the patient.

We selected the fibroids based on the FIGO classification (25). In this classification all myomas are classified into 9 types (0-8): Type 0) a pedunculated submucosal fibroid entirely within the cavity; Type 1) a submucosal fibroid with less than 50% of its diameter within the myometrium; Type 2) a submucosal fibroid ≥50% of the diameter within the myometrium; Type 3) about the endometrium without any intracavitary component; Type 4) intramural and entirely within the myometrium; Type 5) subserosal with at least 50% intramural; Type 6) subserosal with less than 50% intramural; Type 7) subserosal attached to the serosa by a stalk; Type 8) no involvement of the myometrium (cervical lesions, in the round or broad ligaments, "parasitic" fibroids).

The inclusion criteria were as follows: having intra-mural and sub-serosal fibroids, types 3-6. This was because submucosal myomas (types 0-2) are always accompanied by endometrial opening, whereas subserosal pedunculated myomas (type 7) and type 8 myomas do not have any relationship with the myometrium. Surgery (both laparoscopy and laparotomy) was performed under generalized anesthesia. Hysteroscopy was initially performed in order to determine the type of myoma and detect the intra-uterine pathology, if there was any. The myomectomy surgical technique was similar by both laparoscopy and laparotomy. For uterine suturing, the 1-0 vicryl was used. In the case of endometrial opening, 3 layers and in other cases 2 layers were used. Demographic data and myoma characteristics (size, number, type and location) were recorded during surgery. It was also documented whether the endometrium was opened or not. 

Exclusion criteria consisted of a history of uterine surgery (Cesarean, curettage, myomectomy, metroplasty), the presence of systemic conditions which increase the occurrence rate of synechiae such as genital tuberculosis, endometritis or post-operative fever of unknown origin. The probability of post-operative intra-uterine adhesions, its impact on pregnancy or later menstruations, and the benefits of hysteroscopy in the early diagnosis and treatment of this condition were explained for the patients at discharge. If interested in participation in the study, the patients were given the consent form to fill and a due date for undergoing hysteroscopy 3 months later.

After 3 months of surgery hysteroscopy was performed. In case of any adhesions, their severity was determined and then resected. In the next step conjugated Esterogen 2.5 mg daily, for 30 days was prescribed for all patients. In the last 10 days of treatment, a daily regimen of 10 mg medroxyprogesterone acetate was added. Based on the American Society of Reproductive Medicine classification, the adhesions were divided as stage I (mild), II (moderate), or III (severe). This classification is based on the extent of cavity involved, type of adhesion, and menstrual pattern (26). Eventually, the occurrence rate and severity of intra-uterine adhesions and its relationship with type (subserosal, intramural), size (cm), number and location of myoma; and also the opening condition of endometrium were investigated and compared between the two groups.

**Statistical analysis**

The obtained data was analyzed with SPSS software (Statistical Package for the Social Sciences, version 16.0, SPSS Inc, Chicago, Illinois, USA”) using T-test, ^2^, and Anova tests. Confidence interval of 95% was considered statistically significant. P<0.05 was considered as reliable level. 

## Results

A total number of 40 women patients entered the study; 19 cases in the laparoscopy group and 21 cases in the laparotomy group. The number of participants approached to take part in the trial were 57 women, 52 ones were eligible, then we lost 12 of participants, 5 of them did not received myomectomy because of anesthesia contraindications; and 7 ones were lost to follow up because they did not accepted to do hysteroscopy. The mean age of participants in the laparoscopy group was 34.4 yr (Min=27, Max=40), and 35.2 yr (Min=27, Max=48) in the laparotomy group; showing no significant difference between the two groups (p=0.82). The mean parity of the laparoscopy group was 0.2 (Min=0, Max=1) and of the laparotomy group was 0.3 (Min=0, Max=1). The frequency of abortion was 21.1% and 14.3% in the laparoscopy and laparotomy group, respectively. In each group one patient had a history of more than one abortion.

The frequency of patients’ complaints is summarized in table I. The most common symptom in the laparoscopy group was infertility whereas both infertility and AUB were the most common complaint in the laparotomy group. The mean size of myomas in the laparoscopy and laparotomy group was 5.36 cm (min=2, max=10) and 6.38 cm (min=2, max=12), respectively; showing no significant difference (p=0.18). The mean number of myomas in the laparoscopy and laparotomy group was 1.7 (a total of 36), and 1.6 (a total of 34), respectively. The frequency distribution of myomas according to their type (subserosal or intramural) and number is described in table II. The two groups showed no significant difference regarding this issue.

The occurrence rate of synechiae in the laparoscopy group was 21% and was 19% in the laparotomy group (Table III). There was no statistically significant difference between the two groups in the occurrence rate and severity of synechiae (p=0.99). Since no significant difference was revealed between the two groups of laparoscopy and laparotomy in terms of various factors of myoma and synechiae, and as the two groups had a small size; in order to assess the relationship between synechiae with other factors, all participants were evaluated as a single group. Accordingly, the relationship between size, type, location and number of myomas and also the opening of endometrium with the frequency and severity of synechiae were investigated (Table IV).

Synechiae occurred more frequently when the endometrium had been opened (25% vs. 18.8%), but there was no statistically significant difference (p=0.92). Using ANOVA test, a significant relationship was detected between mean myoma size and uterine synechiae (p=0.01). 

This means that with an increase in the size of myoma, synechiae probability would be increased. Accordingly, the Scheffe test showed that this difference is only between the sever forms of synechiae with normal condition (not with mild and moderate synechiae). Among the 8 cases of synechiae, only one had a subserosal myoma which was of the mild type. However, the type of myoma was not significantly related to the frequency and severity of synechiae (p=0.08), despite a p≈0.05. 

Also, the location of myoma was not significantly related to the frequency and severity of synechiae (p=0.14); however, given the location of largest myomas, the probability of adhesion in fundal myomas was significantly higher (p=0.02). In order to evaluate the relationship between the numbers of myomas with uterine synechiae, we wished to use Multinomial logistic test, which was not possible due to the limitation of our sample size. 

Alternatively, the ANOVA test was applied, indicating a significant relationship between them (p=0.03). Then, using the Bonferroni test, the number of myomas showed a positive correlation with the probability of synechiae occurrence, but had no relationship with its severity.

**Table I:** Frequency distribution of patient’s symptoms according to the surgical approach

** Surgery approach**	**Laparoscopy **	**Laparotomy **
**Symptoms**		
Infertility	14 (73.7)	8 (38.1)
Pelvic pain	1 (5.3)	1 (4.8)
AUB	3 (15.8)	8 (38.1)
Recurrent abortion	1 (5.3)	0 (0)
Pelvic mass	0 (0)	3 (14.3)
Urinary symptoms	0(0)	1 (4.8)
Total	19 (100)	21(100)

Data are presented as n (%).

**Table II:** Frequency distribution of myomas’ types according to the surgical approach

**Surgical approach**		**Laparoscopy **	**Laparotomy **	**Total **	**p-value**
Kind of myoma					0.70
	Subserose	17 (47.2)	15 (44.1)	32 (45.7)	
	Intramural	19 (52.8)	19 (55.9)	38 (54.3)	
	Total	36 (100)	34 (100)	70 (100)	
Number of myoma in each patient					0.69
	1	19 (52.8)	21(61.8)	40 (57.2)	
	2	8 (22.2)	10 (29.4)	18 (25.7)	
	3	5 (13.9)	3 (8.8)	8 (11.4)	
	4	4 (11.1)	0	4 (5.7)	
	Total	36 (100)	34 (100)	70 (100)	

Data are presented as n (%).

Two groups had no significant difference using Student’s *t*- test (number of myomas) and  ^2^ test (kind of myomas).

**Table III:** Comparison uterine synechiae between laparoscopy myomectomy group with laparotomy group

** Synechiae**	**Absent **	**Mild to moderate **	**Severe **	**Total **
**Surgical approach**				
Laparoscopy	15 (79)	2 (10.5)	2 (10.5)	19 (100)
Laparotomy	17 (81)	2 (9.5)	2 (9.5)	21 (100)
Total	32 (80)	4 (10)	4 (10)	40 (100)

Data are presented as n (%). p= 0.99

**Table IV:** Investigating the relationship between different characteristics of myoma and the rate and severity of synechiae after myomectomy

** Synechiae**			**Absent **	**Mild to moderate **	**Severe **	**Total **	**p-value**
**Factors related to myoma**							
Endometrial opening							0.92
	Yes		6 (75)	1 (12.5)	1 (12.5)	8 (100.0)	
	No		26 (81.2)	3 (9.4)	3 (9.4)	32 (100.0)	
Size (Mean)			5.9 CI 95%: 1.8-2.5	5.7 CI 95%: 1.6-4.2	5.8 CI 95%: 2.7-4.6		0.03
Kind							0.08
	Subserosal		12 (92.3)	1 (7.7)	0 (0.0)	13 (100.0)	
	Intramural		17 (85)	1 (5.0)	2 (10.0)	20 (100.0)	
	Both		3 (42.9)	2 (28.6)	2 (28.6)	7 (100.0)	
Location of the biggest myoma							0.02
	Fundus		7 (70.0)	2 (20.0)	1 (10.0)	10 (100.0)	
	Anterior		15 (93.75)	1 (6.25)	0 (0.0)	16 (100.0)	
	Posterior		7 (100.0)	0 (0.0)	0 (0.0)	7 (100.0)	
	Right		2 (50.0)	1 (25.0)	1 (25.0)	4 (100.0)	
	Left		1 (33.3)	0 (0.0)	2 (66.7)	3 (100.0)	
Number (Mean)			1.5 CI 95%: 1.2-1.8	2.0 CI 95%: 1.1-2.9	2.8 CI 95%: 1.2-4.3	25 (100)	0.03

Data are presented as n (%).

The relationship between mean myoma size and mean myoma number with synechia, were analysed using ANOVA test; and relationship between endometrial openings, myoma’s kind and location with synechia, were analysed using ^2^ test.

**Figure 1:**
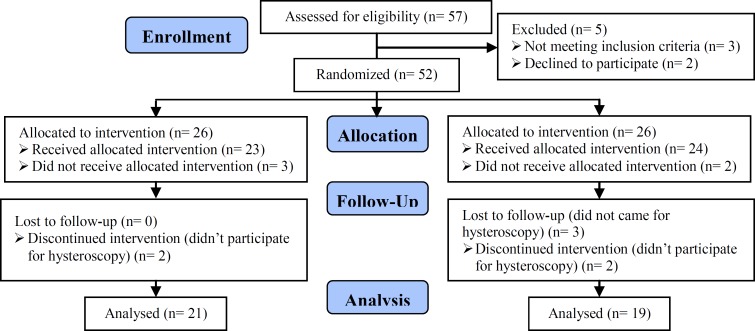
Consort flow chart.

## Discussion

In this study, the most common symptom of patients with myoma was infertility and AUB. The reason might be that we did not include virgin patients, and this was a limitation for our study. According to cultural and legal considerations in Iran, diagnostic or therapeutic procedures which are transvaginal, such as hysteroscopy, are legally banned in virgin women. In order to investigate the occurrence of synechiae in our patients, we performed hysteroscopy 3 months after the operation; this interval was originally proposed by Tixier *et al* in 2010 (24). Myoma parameters (size, number and type) were not statistically different between the two laparoscopy and laparotomy groups. 

The occurrence rate of synechiae was 21% and 19% (mean: 20%) in laparoscopy and laparotomy groups, respectively. Given the appropriate suturing technique in laparoscopy, the probability of endometrial damage would be similar to laparotomy. This was also emphasized in Tulandi and al-took study (27). To note, Tixier *et al* did not separate the two laparoscopy and laparotomy groups to compare the rate of synechiae; they obtained a total occurrence rate of 14.8% for synechiae which was lower than ours (24). In our study, synchiea was more probable when the endometrium was opened, but the relationship was not statistically significant. Gambadauro *et al* in 2012 also found a significantly higher occurrence of synechiae when the endometrium was opened (18). 

However, considering the mechanism of synechiae development after myomectomy, it should be emphasized that even without endometrial opening, there is still a possibility for necrosis and fibrosis in the endometrium (28). In our study, a significant relationship was detected between the mean myoma size and the occurrence of synechiae. This was in agreement with Sizzi *et al* study, which concluded that the size of myoma affects its major complications rate (29). 

We also found a higher occurrence of synechiae in intramural myomas. In our study, only one case with synechiae had a subserosal myoma. However, we did not find a statistically significant relationship which might be due to our limited sample size. Other studies have indicated that synechiae has significantly higher incidence in the intramural group (18, 19). Nevertheless, we also achieved a significant increase in the occurrence of uterine synechiae with a larger number of myomas. This finding is consistent with several other studies (29).

## Conclusion

This study showed that given similar surgical techniques, laparoscopy and laparotomy have a similar risk for intrauterine adhesions formation. We also strongly recommend hysteroscopy to be performed around 3 months after myomectomy as a secondary preventive approach for the diagnosis and treatment of possible synechiae in all cases of myomectomy, even those with subserosal myoma and cases with endometrial opening (despite the possibility of synechia being low in these two groups, 7.7% in subserosal myomas and 18.8% in cases without endometrial opening).

## Note

***Registration ID in IRCT: IRCT2014123020486N1***
